# Expression profiles and transcript properties of fast-twitch and slow-twitch muscles in a deep-sea highly migratory fish, *Pseudocaranx dentex*

**DOI:** 10.7717/peerj.12720

**Published:** 2022-03-30

**Authors:** Huan Wang, Busu Li, Long Yang, Chen Jiang, Tao Zhang, Shufang Liu, Zhimeng Zhuang

**Affiliations:** 1 Yellow Sea Fisheries Research Institute, Chinese Academy of Fishery Sciences, Qingdao, Shandong, China; 2 Laboratory for Marine Fisheries Science and Food Production Processes, Pilot National Laboratory for Marine Science and Technology, Qingdao, Shandong, China; 3 College of Fisheries, Zhejiang Ocean University, Zhoushan, Zhejiang, China; 4 College of Fisheries and Life Science, Dalian Ocean University, Dalian, Liaoning, China; 5 Dalian Tianzheng Industry Co., Ltd., Dalian, Liaoning, China

**Keywords:** *Pseudocaranx dentex*, Fast-twitch muscle, Slow-twitch muscle, Transcriptome expression profiles, Differentially expressed genes

## Abstract

Fast-twitch and slow-twitch muscles are the two principal skeletal muscle types in teleost with obvious differences in metabolic and contractile phenotypes. The molecular mechanisms that control and maintain the different muscle types remain unclear yet. *Pseudocaranx dentex* is a highly mobile active pelagic fish with distinctly differentiated fast-twitch and slow-twitch muscles. Meanwhile, *P. dentex* has become a potential target species for deep-sea aquaculture because of its considerable economic value. To elucidate the molecular characteristics in the two muscle types of *P. dentex*, we generated 122 million and 130 million clean reads from fast-twitch and slow-witch muscles using RNA-Seq, respectively. Comparative transcriptome analysis revealed that 2,862 genes were differentially expressed. According to GO and KEGG analysis, the differentially expressed genes (DEGs) were mainly enriched in energy metabolism and skeletal muscle structure related pathways. Difference in the expression levels of specific genes for glycolytic and lipolysis provided molecular evidence for the differences in energy metabolic pathway between fast-twitch and slow-twitch muscles of *P. dentex*. Numerous genes encoding key enzymes of mitochondrial oxidative phosphorylation pathway were significantly upregulated at the mRNA expression level suggested slow-twitch muscle had a higher oxidative phosphorylation to ensure more energy supply. Meanwhile, expression patterns of the main skeletal muscle developmental genes were characterized, and the expression signatures of *Sox8*, *Myod1*, *Calpain-3*, *Myogenin*, and five insulin-like growth factors indicated that more myogenic cells of fast-twitch muscle in the differentiating state. The analysis of important skeletal muscle structural genes showed that muscle type-specific expression of *myosin*, *troponin* and *tropomyosin* may lead to the phenotypic structure differentiation. RT-qPCR analysis of twelve DEGs showed a good correlation with the transcriptome data and confirmed the reliability of the results presented in the study. The large-scale transcriptomic data generated in this study provided an overall insight into the thorough gene expression profiles of skeletal muscle in a highly mobile active pelagic fish, which could be valuable for further studies on molecular mechanisms responsible for the diversity and function of skeletal muscle.

## Introduction

Skeletal muscle constitutes the largest organ system and is essential for locomotion and body metabolic homeostasis in vertebrate. It is widely accepted that skeletal muscle is composed mainly of two fiber types: slow-twitch and fast-twitch muscle fibers, with different morphological, biochemical, and physiological properties (Frontera & Ochala, 2015). Unlike mammals, the slow-twitch and fast-twitch muscle fibers of fish are spatially segregated into anatomically distinct areas (Berchtold, Brinkmeier & Muntener, 2000). Generally, slow-twitch muscle is restricted to a thin, superficial, lateral wedge in the vicinity of lateral line, while fast-twitch muscle makes up the reminder majority of the muscle bulk (Syme, 2005). This unique distribution pattern makes fish an ideal animal model for investigating the underlying molecular mechanisms that control the diversity and function of skeletal muscle fibers.

Over the past decades, extensive researches have been conducted on teleost skeletal muscle, the classification of muscle fiber types (Kronnie et al., 1983; Silva et al., 2008), the biochemical component distinctions between different muscle types (Gibb & Dickson, 2002), the impact of stress and nutrition on the growth of fast-twitch muscle (Aedo et al., 2015; Magnoni et al., 2015), and the function of single gene in muscle differentiation and development (Chauvigne et al., 2005; Macqueen & Johnston, 2006) were formulated clearly. Recently, limited studies on comparing the gene expression patterns between fast-twitch and slow-twitch muscles of *Takifugu rubripes* (Gao et al., 2017), *Piaractus mesopotamicus* (Mareco et al., 2015), and *Schizothorax prenanti* (Li et al., 2019), have revealed that the complex transcriptional regulatory mechanisms in both metabolic pathways and structural components. However, given the significant correlation between swimming performance, muscle proportion, and energy metabolism (Gibb & Dickson, 2002; Drazen, Dugan & Friedman, 2013; Teulier et al., 2019), studies on these species with relatively weak swimming ability were not enough to characterize the molecular components and regulatory mechanisms that control the muscle types of all teleost, especially for highly athletic species. It is necessary to carry out an accurate and systematic transcriptome research to understand the genetic information responsible for the difference of muscle types on more active fish.

*Pseudocaranx dentex* (Bloch & Schneider, 1801), also known as white trevally, which belongs to the family Carangidae, occurs on continental and island shelves across the anti-tropical regions of the Atlantic, the Indo-Pacific, and the Mediterranean (Smith-Vaniz, 1999). It is a very active and fast swimmer with the habit of pelagic, long-distance migration and has a higher proportion of slow-twitch muscle compared to most other teleost (Rowling & Raines, 2000; Aji, 2011). It has become a target species for finfish aquaculture in Japan and China because of its excellent meat quality and rapidly increasing market demands (Honryo et al., 2021). The main limitation of developing the artificial breeding and aquaculture industry for *P. dentex* is the lack of genetic and physiological information. Therefore, characterization the main skeletal muscle types of *P. dentex* is essential for promoting the development of deep-sea aquaculture industry and the exploitation of long-distance migratory fish resources. Researches on *P. dentex* have mainly focused on the reproductive habitat (Guirao et al., 2005; Afonso et al., 2008), broodstock nutrition (Watanabe & Vassallo-Agius, 2003), seeding production (Nogueira et al., 2018; Honryo et al., 2019), and disease control (Al Bulushi et al., 2010; Imajoh et al., 2013). So far, little was known concerning the expression profiles and transcript properties of fast-twitch and slow-twitch muscles in *P. dentex*.

In this study, we used RNA sequencing technology to capture and compare the transcriptional profiles of fast-twitch and slow-twitch muscles in *P. dentex*. According to the reference genome, the obtained transcriptome data were assembled and the gene expression profiles were investigated. Function annotation and enrichment analysis on the differentially expressed genes (DEGs) were also conducted. This work aims to provide a basis for future studies on molecular mechanisms that responsible for the diversity and function of skeletal muscle in *P. dentex*.

## Materials and Methods

### Ethics statement

All the experimental animal treatment in this study was approved by the Animal Care and Use Committee of Yellow Sea Fisheries Research Institute (Permit No. YSFRI-2021013). All procedures were in strict accordance with the guide for the care and use of laboratory animals and the animal welfare in China.

### Sample collection and RNA extraction

**Figure 1:**
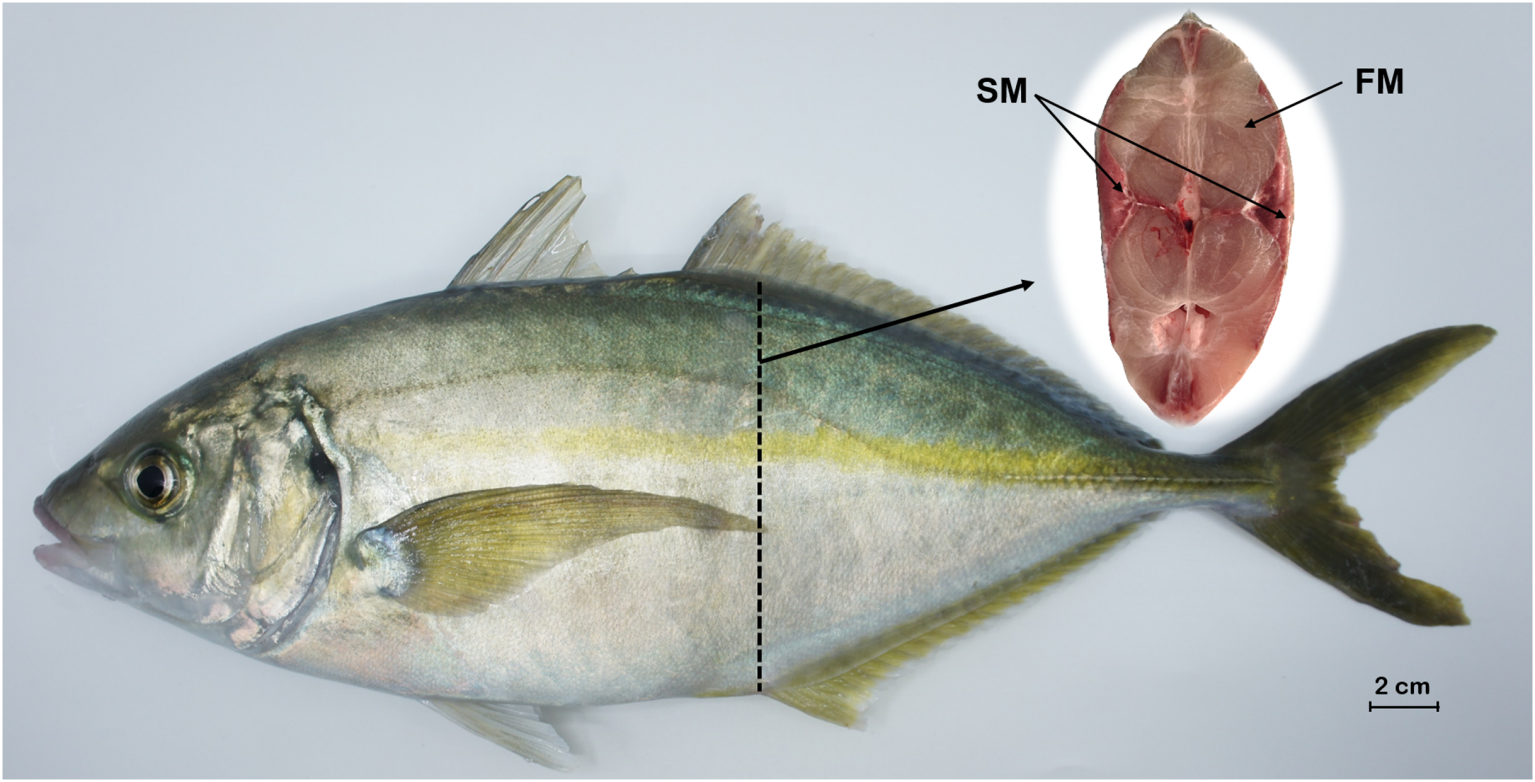
Tissue sampling of *P. dentex* used in this study. The locations of slow-twitch muscle and fast-twitch muscle are marked on the cross section of dashed arrows slice. FM, fast-twitch muscle; SM, slow-twitch muscle. Bar = 2 cm.

A total of three healthy *P. dentex* (body weight: 1567.63 ± 147.05 g; body length: 36.83 ± 0.67 cm) specimens with the same genetic background were randomly collected from the Dalian Tianzheng Industry Co., Ltd. (Dalian, Liaoning province, China) in November 2020. The experimental fish were taken from the same breeding condition (temperature: 15–22 °C; salinity: 23–30; pH: 7.2–8.0; dissolved oxygen: 7–9 mg/L; feed three times a day with fresh fish and artificial diets), which will eliminate the influence of environmental factors. Before the start of sampling, the living *P. dentex* were anesthetized with MS-222 (Tricaine Methanesulfonate) at 30 μg/mL and then euthanased by severing the spinal cord. Fast-twitch muscle was dissected from the dorsal epaxial region, while slow-twitch muscle was dissected from the zone beneath the lateral line region, as shown in Fig. 1. Any ambiguous fiber was removed to obtain pure muscle tissues. Samples were immediately placed into liquid nitrogen and stored at −80 °C until RNA extraction.

Total RNA was extracted from each tissue using RNAiso Plus (Takara, Beijing, China) following the manufacturer’s protocol. The quantity and quality of extracted total RNA were measured using Qubit® 2.0 Fluorometer (Life Technologies, Carlsbad, CA, USA) and 1% agarose gel electrophoresis.

### cDNA library construction and transcriptome sequencing

Sequencing libraries were generated using NEBNext®Ultra™ RNA Library Prep Kit for Illumina® (NEB, Ipswich, MA, USA) and 3 μg total RNA per sample was used as input material following the manufacturer’s recommendation. To attribute sequences, the index codes were added. Three fast-twitch muscle cDNA libraries and three slow-twitch muscle cDNA libraries were constructed. The prepared cDNA libraries were sequenced on the Illumina NovaSeq 6000 platform and 150 bp paired-end reads were generated at Novo Gene Biological InfoTech Ltd. (Beijing, China). The raw data were deposited into NCBI Sequence Read Archive (SRA) with accession numbers SRR14672383, SRR14672384, SRR14672385, SRR14672386, SRR14672387 and SRR14672388.

### RNA-Seq analysis

Raw data were firstly processed through fastP program (version 0.19.7; Chen et al., 2018), which invoked by Perl scripts to remove low-quality reads. Reads containing adapter and ploy-N were also filtered. After initial processing, Q20, Q30, and GC content were calculated. Then, the clean reads were aligned to the *P. dentex* reference genome (NCBI: PRJNA731999) using TopHat v2.0.12 (-max-intron-length 500000 -m 2 --library-type fr-unstranded) (Trapnell, Pachter & Salzberg, 2009) with Bowtie as internal aligner. The reads numbers mapped to each gene were counted with HTSeq v0.6.1 (union mode) (Anders, Pyl & Huber, 2015). FPKM, which considers the effect of gene length and sequencing depth for the reads count at the same time, was calculated for estimating gene expression levels based on the formula: FPKM = 10^9^ × C/(N × L), where, C is the number of reads mapped onto each gene, N is the total number of mapped reads onto all genes, and L is the base length of gene. When FPKM value > 1.0, the gene was considered to be expressed. Pearson correlation matrix and principle component analysis (PCA) were performed to assess the reproducibility and variability of biological samples. Upon the expression levels of all genes, the Pearson’s correlation coefficient (r) was calculated with the R function ‘correl’ and PCA on all samples were performed using TBtools. Generally, when |r| ≥ 0.8, it was regarded as a high correlation between the two variables; when 0.5 ≤ |r| < 0.8, it was regarded as moderate correlation; when 0.3 ≤ |r| < 0.5, it was regarded as low correlation; when |r| < 0.3, it was regarded as irrelevant between the two variables.

We used DESeq to find genes that differentially expressed between fast-twitch muscle and slow-twitch muscle. The significance of the differential gene expression was determined by the control of false discovery rate (FDR) calculated based on Benjamini and Hochberg method (Benjamini & Hochberg, 1995). Genes with both fold change ≥2 and FDR adjusted *p* < 0.05 were assigned as differentially expressed (Anders & Huber, 2012). The volcano plot and hierarchical clustering heatmap to present the global distribution of differentially expressed genes (DEGs) were graphed using pheatmap R package and ggplot2 R package, respectively. To determine the functions of DEGs, Gene Ontology (GO) enrichment analysis by GOseq R package based on Wallenius non-central hypergeometric distribution (Young et al., 2010), and KEGG (http://www.genome.jp/kegg/) (Kanehisa et al., 2008) pathways annotation by KOBAS software (Mao et al., 2005) were conducted. The chord plot was generated with the GOplot R package (https://cran.r-project.org/web/packages/GOplot) (Walter, Sánchez-Cabo & Ricote, 2015).

### Validation of gene expression profiles by RT-qPCR analysis

RT-qPCR analysis was used to validate the gene expression changes observed by RNA-seq. A total of 12 DEGs was selected for validation and *GAPDH* was used as the internal reference gene, as shown in Table 1. The same subset of RNA samples from fast-twitch muscle and slow-twitch muscle used in RNA-seq were analyzed. RT-qPCR was carried out using SYBR Green Pro Taq HS Mix (Accurate Biotechnology, Hunan, China) and performed in a 7500 Fast Real-Time PCR System (Applied Biosystems, Waltham, MA, USA). The amplification efficiency of each primer pair was verified using a cDNA dilution series. Relative expression values were determined using 2^−ΔΔCt^ method (Livak & Schmittgen, 2001). The Pearson correlation coefficient was used to investigate the correlation between RNA-seq and RT-qPCR results.

**Table 1:** Primers of DEGs used for gene expression changes validation with RT-qPCR method.

NO	Gene ID	Gene name	Log_2_ (fold change)	*p-*value	Primers sequences (5′-3′)
1	evm.TU.Hic_asm_5.399	Glyceraldehyde-3-phosphate dehydrogenase (*GAPDH*)	0.65252	0.041328	F: TTGGTTACAGCCACCGTGTT R: GCTATGGATGGGGCTTGTGT
2	evm.TU.Hic_asm_1.284	Myoblast determination protein 1 (*Myod1*)	−6.8263	5.39E−16	F: ACGACAACGGCTTCTACCCTC R: TCTGTGCTGATCCGCTCTACG
3	Novel00281	Myosin7 (*Myo7b*)	9.7529	1.38E−29	F: CCGGGCTTTCATGGGAGT R: CCTGCGGGCTTCTGATTTT
4	evm.TU.Hic_asm_1.938	Myosin-binding protein C, cardiac-type (*MybpC3*)	9.6835	1.76E−19	F: GATTGAAGGCGTGCCGTAT R: CACTCGTAGGAGCGACTGG
5	evm.TU.Hic_asm_10.313	Myosin light chain 1, skeletal muscle isoform (*MyLC1*)	−6.9523	1.72E−17	F: GGCGGTATCAACTACGAGGG R: GTATACAATGGGTGCACTGCC
6	evm.TU.Hic_asm_19.316	Myosin regulatory light chain 2, skeletal muscle isoform (*MyL2*)	−7.3122	2.35E−14	F: TCACTGTGTGAGAGCAACCAA R: GGAGCGGAGAGAAAGAGATCG
7	evm.TU.Hic_asm_11.714	Myosin regulatory light chain 2B (*MyL2b*)	9.6208	8.88E−112	F: TGAAAGAAGCTCCAGGTCCA R: TTGTCCTCTCCGTGGGTGATAA
8	evm.TU.Hic_asm_3.435	Myosin regulatory light chain 2, atrial isoform (*MyL7*)	17.02	1.93E−07	F: AAGACTTGAGGGAGACGTATGG R: TGATGTAGCAGAGCGACTTGTAG
9	evm.TU.Hic_asm_9.194	Troponin T, fast skeletal muscle isoforms (*TNNT2*)	−7.0357	6.27E−27	F: AGTAAATTCAGCAAGAAGGGAGC R: TGCATGTATCAGGACGTGGG
10	evm.TU.Hic_asm_22.520	Troponin T, cardiac muscle isoforms (*TNNT3*)	9.2931	7.41E−71	F: GCAGAAGTTCGCCAAAGGAAG R: TTTATGTGTACGCCCAGCTCT
11	evm.TU.Hic_asm_2.756	ADP/ATP translocase 1 (*SLC25A4*)	−6.141	7.34E−17	F: GTCGTGACCTGGATGATTGC R: TTCCTTAGGGACTGGGAGATTAG
12	evm.TU.Hic_asm_12.1071	Calsequestrin-1 (*CASQ1*)	−7.2504	1.59E−44	F: GAAGTACGATGTCATGGTGGTG R: CAATGAGACCGACTCCGATA
13	evm.TU.Hic_asm_7.23	Troponin C, slow skeletal and cardiac muscles (*TNNC1*)	7.3002	1.46E−76	F: CTCTCTGCAACAGCTCTCATCA R: CGAGGACGGGTGTGTTGTTG

## Results

### Sequencing and transcriptome annotations

**Figure 2:**
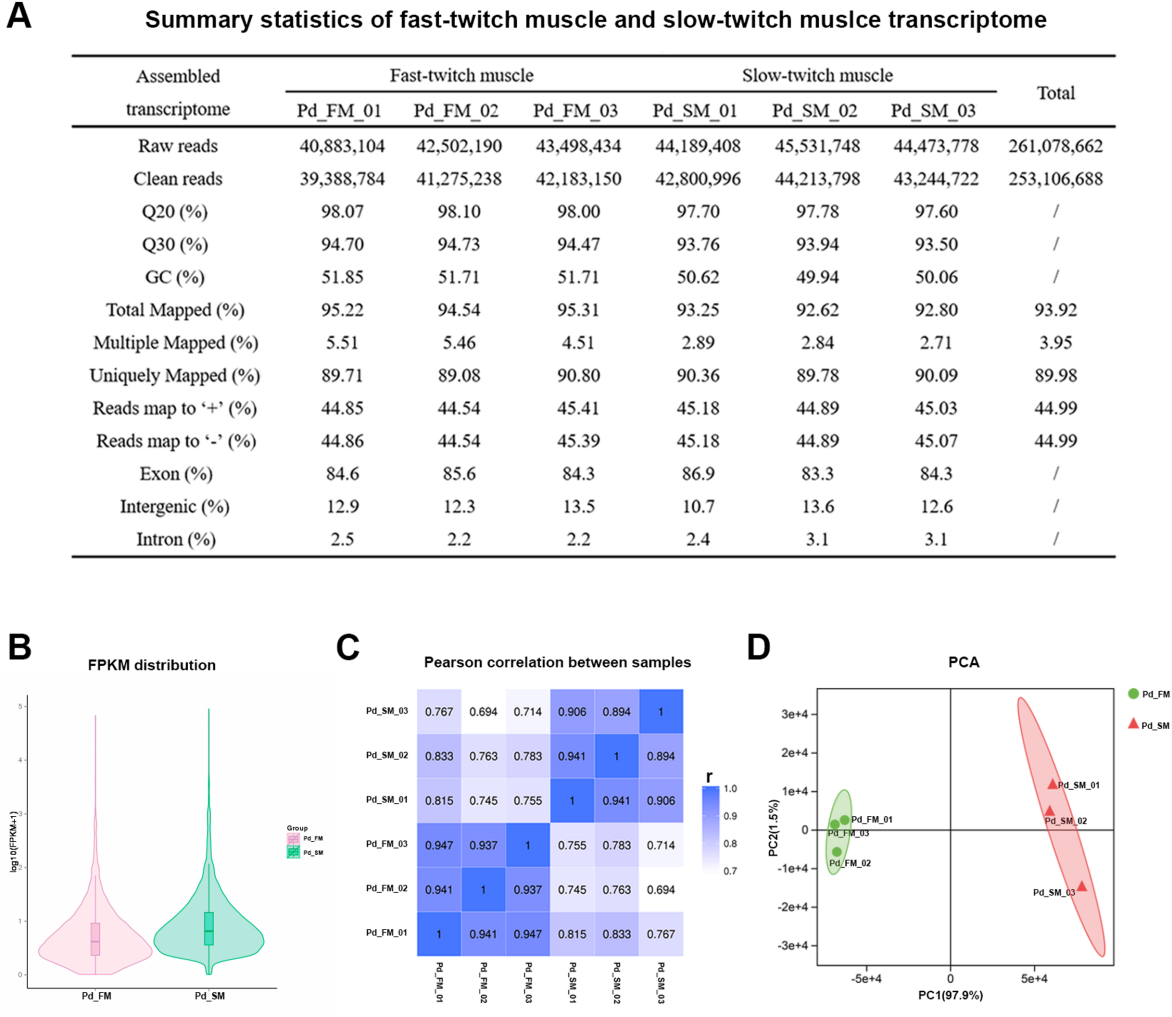
Summary statistics of the *P. dentex* fast-twitch and slow-twitch muscles transcriptome. (A) Statistics summary for Illumina short reads information of transcriptome. (B) FPKM distribution of genes. (C) Pearson’s correlation coefficients between samples. “r” Represents the Pearson’s correlation coefficient. (D) The PCA plot of samples used in current study. Pd_FM, fast-twitch muscle; Pd_SM, slow-twitch muscle.

Here, six separate Illumina sequencing libraries produced close to 261.08 million paired-end reads. After low-quality sequencing reads filtering, a total about 253.11 million high-quality clean reads were obtained: 122.85 million reads from three fast-twitch muscle samples and 130.26 million reads from three slow-twitch muscle samples (Fig. 2A). Q20 varies from 97.60% to 98.10% and Q30 varies from 93.50% to 94.73%. Over 92.62% of the clean reads from all sequencing libraries could be mapped onto *P. dentex* reference genome, and 83.3–86.9% of the reads were mapped to the gene coding regions, which indicated that we have obtained the high-quality transcriptome sequencing data of *P. dentex* skeletal muscle.

In all six samples, 48.54–64.31% of the 24,636 mapped genes detected to be expressed (Table S1). The FPKM distribution between fast-twitch and slow-twitch muscles of *P. dentex* was not completely the same, indicating that overall gene expression level was different (Fig. 2B). The values of correlation among samples intra types of muscle in this study had very high repeatability (r = 0.894–0.947), greater than that of inter types of muscle (r = 0.694–0.833, Fig. 2C). Result of PCA was consistent with Pearson’s correlation analysis. The first two principal components explained 97.9% and 1.5% of the total genetic variances, respectively (Fig. 2D). The PC1 clearly separated the biological samples of fast-twitch muscle from the ones of slow-twitch muscle and grouped the same type of samples together. Both two analyses confirmed the reliability of the experimental process and rationality of sample selection.

### Identification of differentially expressed genes (DEGs)

**Figure 3:**
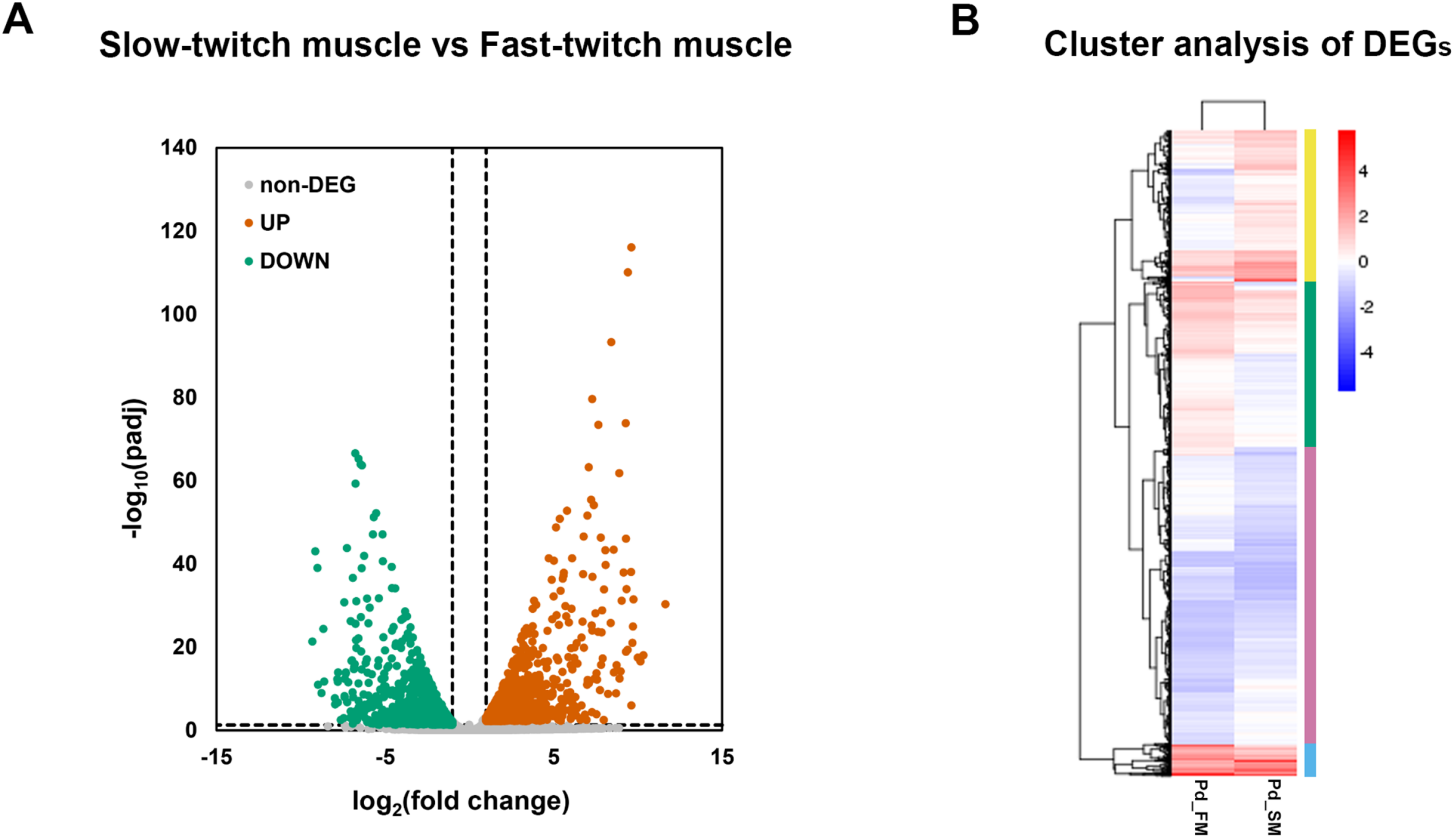
The expression profiles of DEGs in fast-twitch and slow-twitch muscles. (A) The volcanic diagrams visualizing the distribution of DEGs. Under the criteria of fold change ≥2 and *p* < 0.05, up-regulation genes in slow-twitch muscle are represented by red dots, while up-regulation genes in fast-twitch muscle are represented by green dots. (B) Heatmaps of the DEGs between fast-twitch and slow-twitch muscles, which clustering into four groups based on their expression profiles. The columns represent two types of muscle. The rows represent DEGs. The color bars at right represent different gene groups. The expression levels of genes are represented by the value of normalization transformed log_10_ (FPKM + 1). Pd_FM, fast-twitch muscle; Pd_SM, slow-twitch muscle.

A total of 2,862 genes were identified as differentially expressed genes (DEGs) between fast-twitch and slow-twitch muscles, of which 1,419 were significantly higher expressed in slow-twitch muscle and 1,443 in fast-twitch muscle (Fig. 3A). Genes with the same or similar expression patterns were clustered together, implied that these DEGs may perform similar biological functions or participate in the same biological process (Fig. 3B).

### GO enrichment analysis of DEGs

GO enrichment analysis was used to determine the main biological functions of the DEGs. After corrected by FDR, the GO term with *q*-value ≤ 0.05 was defined as significantly enriched. A total 2,558 DEGs were enriched in 3,111 GO terms, and of these 121 GO terms were significantly enriched (*q* ≤ 0.05). The DEGs that up-regulated in slow-twitch muscle were significantly enriched in the mitochondrion (GO:0044429; GO:0005739; Fig. 4A), mitochondrial membrane (GO:0005743; GO:0031966; Fig. 4A), and mitochondrial energy metabolism (GO:0005746; GO:0003824; GO:0016491; GO:0003995; GO:0015002; Fig. 4A) (*q* ≤ 0.05). Although there were no significant enrichment terms of up-regulated DEGs in fast-twitch muscle, the GO analysis still provided us with some information about gene function classification and cellular localization. For example, GO results showed that the DEGs up-regulated in fast-twitch muscle were enriched in skeletal muscle component (GO:0015629; GO:0005861; GO:0005865; GO:0030016; GO:0030017; GO:0036379; GO:0043292; Fig. 4B) and proteolysis catabolic (GO:0051603; GO:0030163; Fig. 4B) (*q* > 0.05).

**Figure 4:**
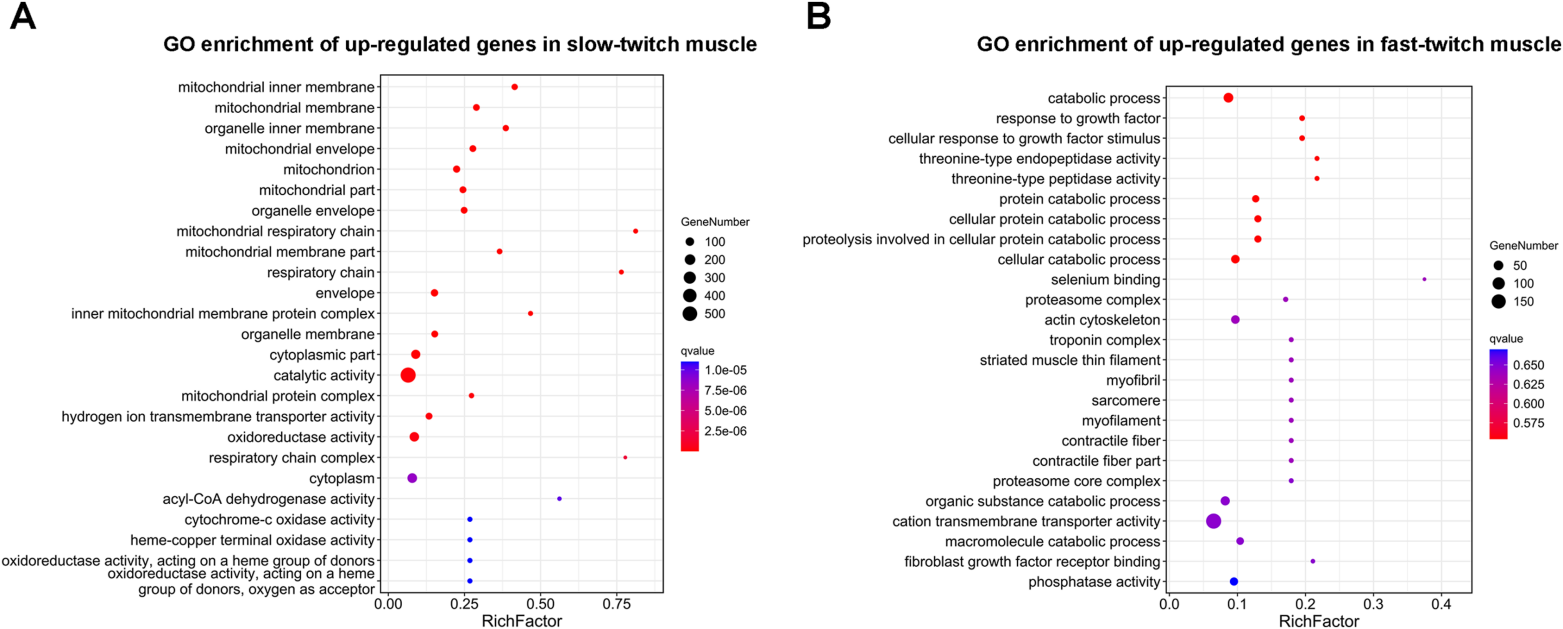
GO enrichment analysis of DEGs between slow-twitch and fast-twitch muscles. (A) The top 25 GO terms ranked by *q*-value of up-regulated genes in slow-twitch muscle. (B) The top 25 GO terms ranked by *q*-value of up-regulated genes in fast-twitch muscle. Rich factor of x-axis indicates the number of enriched genes associated with the given GO term divided by the total number of input genes. The specific terms plotting along the y-axis. The size of the colored dots indicates the number of significantly DEGs associated with each corresponding term. The color of each dot indicates the corrected *q*-value for the corresponding term.

### KEGG enrichment analysis of DEGs

**Figure 5:**
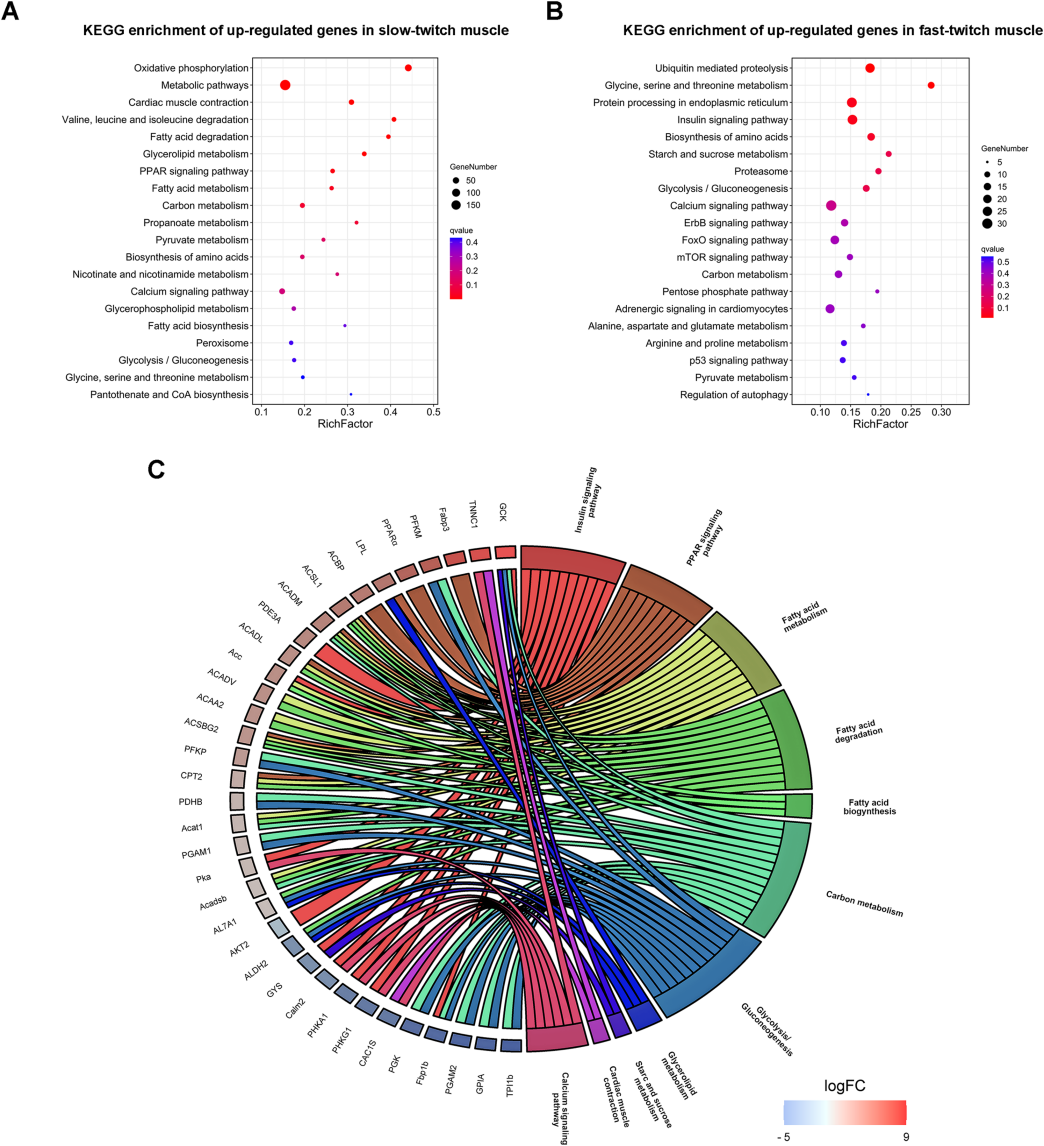
KEGG enrichment analysis of DEGs between slow-twitch and fast-twitch muscles. (A & B) The top 20 GO terms ranked by q-value of up-regulated genes in slow-twitch and fast-twitch muscle, respectively. Rich factor of x-axis indicates the number of enriched genes associated with the given KEGG term divided by the total number of input genes. The specific terms plotting along the y-axis. The size of the colored dots indicates the number of significantly DEGs associated with each corresponding term. The color of each dot indicates the corrected *q*-value for the corresponding term. (C) Chord plot showing the DEGs shared by two or more energy metabolism related KEGG pathways. DEGs on the left ordered by log_2_ (FoldChange). When the gene is more expressed in slow-twitch muscle, the redness of the rectangular flanks is deeper, on the contrary, the blue is darker. Different KEGG pathways on the right shown in different colors.

KEGG pathway analysis was also carried out to categorize the DEGs in metabolic or signal transduction pathways. A total of 147 pathways were identified, and of these only 12 KEGG pathways were significantly enriched (*q* ≤ 0.05). Oxidative phosphorylation (dre00190), cardiac muscle contraction (dre04260), glycerolipid metabolism (dre00561), PPAR signaling pathway (dre03320), and fatty acid degradation (dre00071) were the main pathways in slow-twitch muscle (Fig. 5A). Ubiquitin mediated proteolysis (dre04120), insulin signaling pathway (dre04910), starch and sucrose metabolism (dre00500), and glycolysis/gluconeogenesis (dre00010) were the key pathways in fast-twitch muscle (Fig. 5B). The annotation results of DEGs provided molecular evidence for the difference of energy metabolism between slow-twitch and fast-twitch muscles.

To obtain the specific molecular characteristic information contribute to the differences in energy metabolism, we analyzed the DEGs that control the activity and expression of key enzymes, transporters, and transcription factors in biosynthesis, uptake, and metabolism of lipid and glycogen. Several key genes that participate in lipogenesis (acetyl-CoA carboxylase (*Acc*) and acyl-CoA-binding protein (*ACBP*)), lipid uptake (fatty acid-binding protein (*Fabp3*) and lipoprotein lipase (*LPL*)), and oxidation (peroxisome proliferator-activated receptor α (*PPARα*), acyl-CoA dehydrogenase (*Acadm*), long-chain specific acyl-CoA dehydrogenase (*Acad1*), long-chain fatty acid-CoA ligase (*ACS*), cGMP-inhibited 3′,5′-cyclic phosphodiesterase (*PDE3A*), and cAMP-dependent protein kinase (*PKA*)) were all transcriptionally up-regulated in slow-twitch muscle (Fig. 5C). On the contrary, the key enzymes that control glycolysis/gluconeogenesis, including serine/threonine-protein kinase (AKT), fructose-1,6-bisphosphatase (Fbp1b), phosphoglycerate kinase (PGK), glucose-6-phosphate isomerase (GPIA), and glycogen synthase (GYS) were all up-regulated in fast-twitch muscle at transcriptional level (Fig. 5C).

### Comparative analysis of mitochondrial oxidative phosphorylation related genes

**Figure 6:**
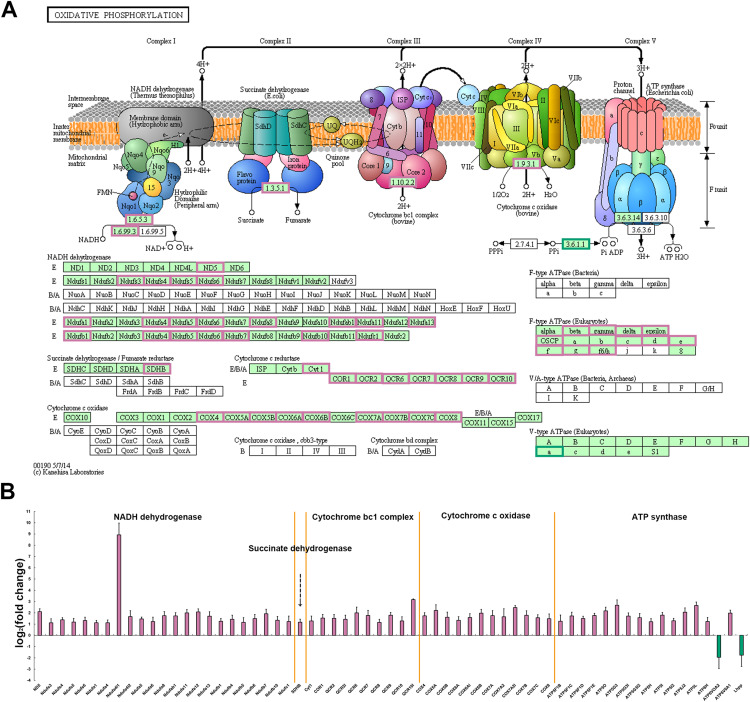
DEGs related to mitochondrial oxidative phosphorylation pathway in *P. dentex* slow-twitch muscle and fast-twitch muscle. (A) DEGs mapping onto the KEGG pathway, which represents oxidative phosphorylation (adapted from KEGG ID: dre00190). The background color of rectangle represents species specificity pathway, “green background” represents eukaryotes, whereas “white background” represents bacteria or archaea. The “reddish purple border” of rectangle represents up-regulated gene in slow-twitch muscle and “bluish green border” represents up-regulated gene in fast-twitch muscle. (B) The fold change on a log2 scale for the genes marked in (A) “Reddish purple bar” indicates up-regulated gene in slow-twitch muscle. “Bluish green bar” indicates up-regulated gene in fast-twitch muscle.

Among all the metabolic pathways, mitochondrial oxidative phosphorylation is the main energy supply process for muscle contraction and is closely linked to the normal function of skeletal muscle (Chabi et al., 2008; Crane et al., 2010; Park et al., 2014). Oxidative phosphorylation process produces and accumulates ATP through electron flow and proton gradient between five protease complexes (complex I-V) located in the inner mitochondrial membrane (Chaban, Boekema & Dudkina, 2014). In *P. dentex* skeletal muscle, a total of 145 genes were annotated into this pathway and among these, 63 DEGs including members of NADH dehydrogenase, succinate dehydrogenase, cytochrome bc1 complex, cytochrome c oxidase, and ATP synthase, have been verified to be significantly differentially expressed between these two muscle types (*p* < 0.05, Fig. 6). *ATP6V0A2* and *Lhpp*, members of the complex V, were the only two genes that up-regulated in fast-twitch muscle, while the other 61 genes showed a higher expression level in slow-twitch muscle (*p* < 0.05, Fig. 6).

### Comparative analysis of skeletal muscle developmental and structural related genes

**Figure 7:**
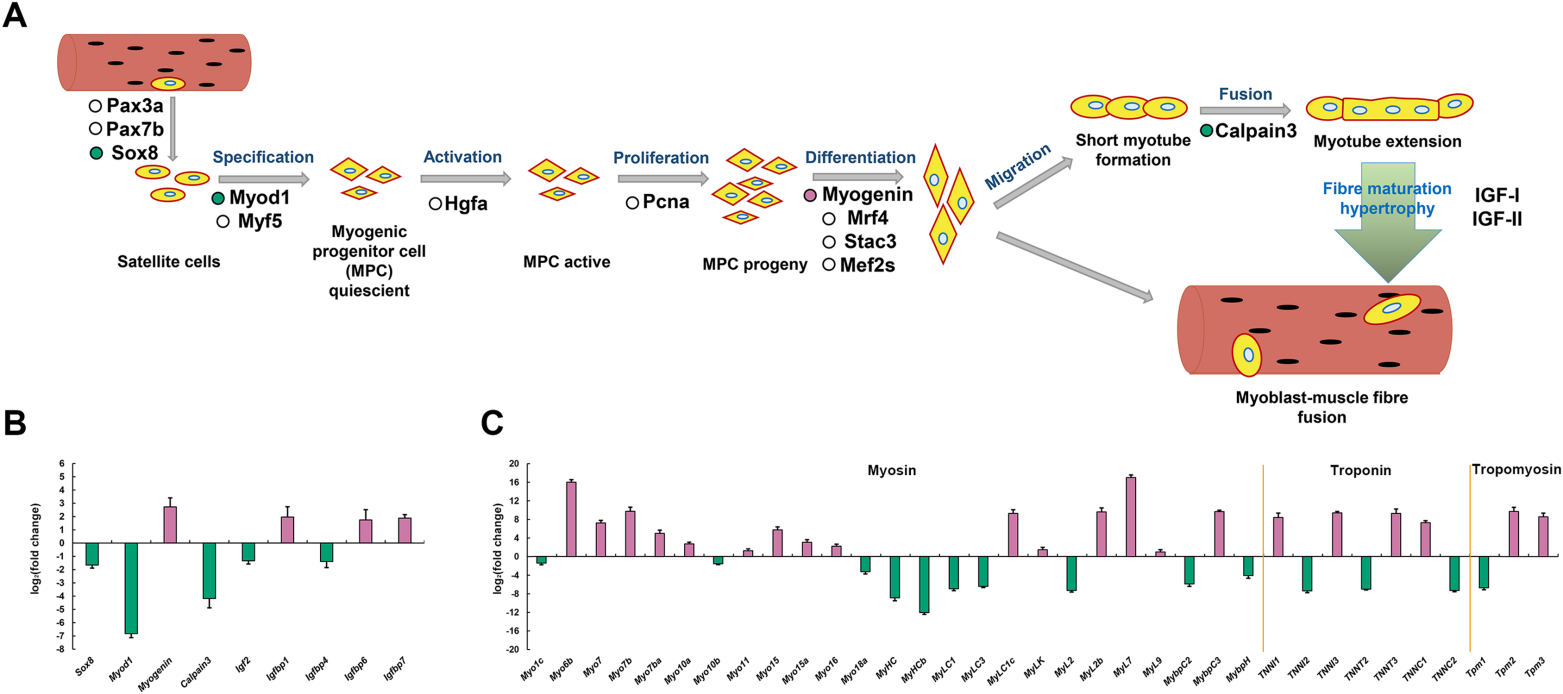
Simplified diagram of skeletal myogenesis and expression profiles of myogenic related genes in *P. dentex* slow-twitch and fast-twitch muscle. (A) The representative myogenic-related genes represented in the transcriptome are mapping into the myogenesis process schematic diagram. “Reddish purple dot” indicates gene significantly up-regulated in slow-twitch muscle. “Bluish green dot” indicates gene significantly up-regulated in fast-twitch muscle. “White dot” indicates gene with no significant differences between two types of muscle. (B) Skeletal myogenesis related genes and (C) Myofibril related genes expression levels. “Reddish purple bar” indicates gene significantly up-regulated in slow-twitch muscle. “Bluish green bar” indicates gene significantly up-regulated in fast-twitch muscle.

To explore the molecular mechanisms of skeletal muscle development and growth in *P. dentex*, a simplified diagram of skeletal myogenesis was drawn based on the existing studies of other vertebrates (Johnston, Bower and Macqueen, 2011; Valente et al., 2013; Frontera & Ochala, 2015) (Fig. 7A). The relative expression levels of key genes associated with myoblast specification, activation, proliferation, differentiation, migration, fusion, and maturity were analyzed (Fig. 7B). According to our RNA-seq data, the genes encoding transcription factor Sox-8 (*Sox8*), myoblast determination protein 1 (*Myod1*), Calpain-3, insulin-like growth factor II (*Igf2*), and IGF-binding protein 4 (*Igfbp4*) were significantly up-regulated in fast-twitch muscle, whereas *Myogenin*, *Igfbp1*, *Igfbp6* and *Igfbp7* were significantly up-regulated in slow-twitch muscle (Fig. 7B). Similarly, genes encoding skeletal muscle structural proteins and related regulatory proteins, such as myosin, myosin heavy chain, myosin light chain, myosin regulatory light polypeptides, myosin-binding proteins, troponin and tropomyosin also showed differentiated expression patterns between slow-twitch muscle and fast-twitch muscle (Fig. 7C).

### RT-qPCR validation

**Figure 8:**
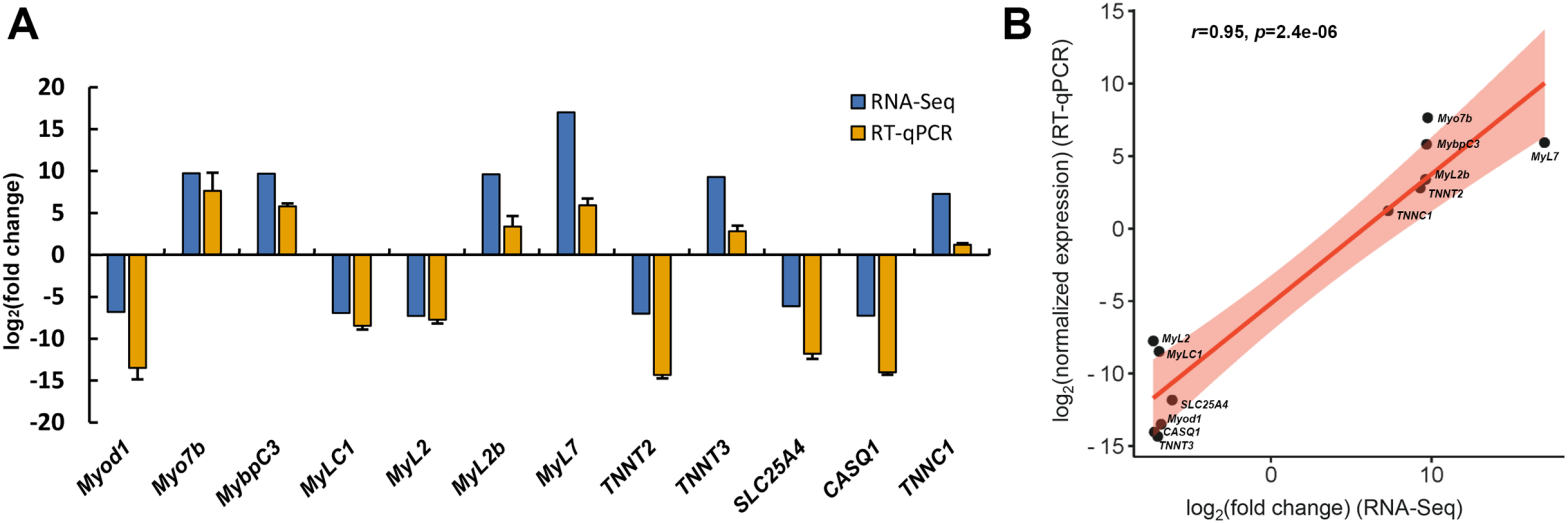
RT-qPCR verification of RNA-seq gene expression profiles. (A) Relative expression profiles of twelve genes when slow-twitch muscle compare to fast-twitch muscle. The GAPDH serving as the reference for quantitative analysis. (B) Correlation analysis of the expression data from RNA-Seq and RT-qPCR. r, Pearson’s correlation coefficient used to reflect the degree of linear correlation between RNA-Seq and RT-qPCR. *p*, *p*-value of the significance level.

To assess the reliability of transcriptome data, twelve DEGs associated with skeletal muscle contraction, including *Myod1*, *Myo7b*, *MybpC3*, *MyLC1*, *MyL2*, *MyL2b*, *MyL7*, *TNNT2*, *TNNT3*, *SLC25A4*, *CASQ1* and *TNNC1*, were selected for RT-qPCR analysis. Among these, six DEGs were significant up-regulated in slow-twitch muscle, while the others were significant up-regulated in fast-twitch muscle according to the RNA-seq data (Table 1). *GAPDH* was used as the reference for quantitative analysis, as its expression levels were confirmed to be consistent between these two types of muscle. The relative expression levels of DEGs obtained by RT-qPCR were in great agreement with the results of transcriptome analysis (Pearson’s *r* = 0.95, *p* = 2.4e−06; Fig. 8), which indicated that the accuracy and reliability of the methods and results presented in the study.

## Discussion

Among teleosts, slow-twitch muscle mainly provides power for sustained swimming, while the fast-twitch muscle is mostly involved in burst swimming or sprinting (Videler, 1993). These two types of skeletal muscles have obvious differences in metabolic and contractile phenotypes (Ciciliot et al., 2013). To compare and determine the underlying molecular mechanisms that control and maintain the different muscle types, we used RNA sequencing technology to identify skeletal muscle type-specific gene expression profiles of *P. dentex*, a highly mobile active pelagic fish with distinctly differentiated slow-twitch muscle and fast-twitch muscle. As the reference genome was available, we obtained 24,636 genes from a range of transcripts, described the expression patterns and predicted the functions of DEGs in detail.

Skeletal muscle requires vast amounts of energy, both for activity and growth. Previous studies have reported that lipids and glycogen are the two main energy donors utilized by muscle tissue to produce ATP (Romijn et al., 1993). Lipid packs more energy per gram than any other fuel to support endurance swimming and also provides most of the ATP for muscle recovery (Richards, Heigenhauser & Wood, 2002; Weber et al., 2016), while glycogen metabolism rapidly produces energy for quickly and forcefully muscle contraction during high-intensity activities (Bao et al., 2020). The energy obtained from lipid or glycogen through the process of *β*-oxidation and glycolysis, respectively. These processes are both dependent on substrate availability and enzymatic pathways (Eaton, 2002; Sylow et al., 2017). The test of energy donor content have proved that slow-twitch muscle has a high content of lipid, whereas fast-twitch muscle with a high level of glycogen (Davison & Goldspink, 1984; Kiessling, Ruohonen & Bjørnevik, 2006). The same is true for related enzyme activities (Knox, Walton & Cowey, 1980; Westerblad, Bruton & Katz, 2010; Sun et al., 2016). In the present study, *ACC*, *ACBP*, *Fabp3*, *LPL*, *PPARα*, *Acadm*, *Acad1*, *ACS*, *PDE3A* and *PKA*, which proved to be directly or indirectly involved in the regulation of skeletal muscle fatty acid uptake and *β*-oxidation process, showed the increased mRNA expression level in slow-twitch muscle compared to fast-twitch muscle. While the expression levels of specific glycolytic genes, such as *AKT*, *Fbp1b*, *PGK*, *GPIA*, and *GYS* were much higher in the fast-twitch muscle of *P. dentex* than in the slow-twitch muscle. Thus, our transcriptome results provide molecular evidence for the differences in energy metabolism between fast-twitch and slow-twitch muscles of *P. dentex*.

Mitochondrial oxidative phosphorylation (OxPhos) is the main energy production pathway, provides most of the ATP for both locomotion and growth. The process of ATP generation and accumulation is performed by means of electron flow and proton gradient between five protein enzyme complex (complex I–V) in the inner mitochondrial membrane (Chaban, Boekema & Dudkina, 2014). Our comparative transcriptome analysis showed that the mRNA expression levels of numerous key enzymes involved in electron transport and oxidative phosphorylation were significantly upregulated in slow-twitch muscle compared to fast-twitch muscle, including several subunits of the Complex I (NADH dehydrogenase), II (succinate dehydrogenase), III (cytochrome bc1 complex), IV (cytochrome c oxidase), and V (ATP synthase) (Fig. 6). Through the up-regulated of NADH dehydrogenase and succinate dehydrogenase complex expression levels, more electrons carried by NADH and FADH_2_ might be passed to upregulated cytochrome bc1 complex and cytochrome C oxidase, and finally reached the terminal electron acceptor O_2_ (Chaban, Boekema & Dudkina, 2014). At the same time, more ATP are synthesized by upregulated ATP synthase using the proton gradients across the inner mitochondrial membrane generated by electron transport (Chaban, Boekema & Dudkina, 2014). Therefore, it was inferred that the higher oxidative phosphorylation to ensure more energy supply for slow-twitch muscle to maintain normal function and development compared to fast-twitch muscle in *P. dentex*, which was supported by previous studies in other fishes such as *T. rubripes*, *P. mesopotamicus*, and *Thunnus orientalis* (Mareco et al., 2015; Gao et al., 2017; Ciezarek et al., 2020).

The specific contractile and metabolic phenotypes of fast-twitch muscle and slow-twitch muscle closely related to the genetic programs that determine the muscle fiber development and growth. Compared to other vertebrates, a unique feature of teleost is that the produce of muscle fiber will continue until reach 40–50% of maximum body length, and growth can take place all lifelong (Greer-Walker, 1970; Higgins & Thorpe, 1990; Johnston et al., 2011). We analyzed the expression patterns of markers involved in myoblast specification, activation, proliferation, differentiation, migration, fusion, and maturity (Fig. 7A). *Sox8* has been proposed to be a molecular marker of satellite cells and acts as a negative regulator of muscle differentiation by maintaining satellite cells at quiescent state (Schmidt et al., 2003). *Myod1* and *Myogenin* both belong to the myogenic regulatory factors (MRFs) family. *Myod1* is responsible for initiating the myogenic program, while *Myogenin* appears later and required for myoblast fusion, myotube formation, and adult muscle fiber differentiation (Rescan, Gauvry & Paboeuf, 1995; Cornelison et al., 2000; Bower & Johnston, 2010). The inverse expression pattern of *Myod1* and *Myogenin* have also been found in other teleosts, such as *Oncorhynchus mykiss* (Rescan, Gauvry & Paboeuf, 1995), *Sparus aurata* (Tan & Du, 2002), and *Paralichthys olivaceus* (Zhang et al., 2006). *Calpain-3* is a calcium-dependent cysteine protease that maintains the integrity of sarcomere by regulating the turnover of sarcomeric protein and induces the proteolysis of *Myod1*, and promotes the generation of a pool of reserve cells (Stuelsatz et al., 2010). Therefore, the higher expression level of *Sox8*, *Myod1* and *Calpain-3* may reflect the more extensive development of satellite cells and myogenic cells in *P. dentex* fast-twitch muscle, which are required to achieve shorter contraction cycles during high-speed swimming (Fleming et al., 1990). While the higher expression level of *Myogenin* in slow-twitch muscle suggested that more myoblasts are in the cell fusion phase to form T-tubules and sarcoplasmic reticulum for continuous swimming.

It was known that skeletal muscle growth is directly stimulated by insulin-like growth factors (IGF) through proliferation, differentiation, hypertrophy, and protein synthesis (Hoppeler, 2016). The IGF pathway consists of multiple IGF ligands, IGF receptors, and IGF-binding proteins. In *P. dentex*, five genes (*Igf2*, *Igfbp1*, *Igfbp4*, *Igfbp6*, and *Igfbp7*) showed significant expression difference at mRNA level between slow-twitch muscle and fast-twitch muscle (Fig. 7B). Both *in vivo* and *in vitro* studies have shown that *Igf2* plays a critical role in promoting myogenesis, and its expression level increase dramatically during myogenesis stage (Florini et al., 1991; Ren, Accili & Duan, 2010). The effective concentration and delivery of *Igf2* was regulated through the interaction with IGF-binding proteins, *Igfbp4* was one of them (Sara & Hall, 1990). In addition, it was reported that *Igfbp4* has the effect of enhancing cardiomyocyte differentiation (Zhu et al., 2008). It is possible that the higher abundance of *Igf2* and *Igfbp4* in *P. dentex* fast-twitch muscle is related to the promotion of myoblast differentiation. *Igfbp1*, *Igfbp6*, and *Igfbp7* were the both negative regulators of IGF ligands’ actions, and might have inhibitory roles in fish muscle growth (Fuentes et al., 2013). To some extent, the differential expression patterns of these five genes between the two muscle types of *P. dentex* indicated that there are more myogenic cells in the differentiating state in fast-twitch muscle, compared to slow-twitch muscle.

Myofibrillar proteins, such as myosin, tropomyosin, and troponin, is the predominant component of protein in skeletal muscle, important for maintain the structure of skeletal muscle fibers, excitation-contraction processes, force generation, and energy release (Ottenheijm & Granzier, 2010). Myosin, one of the most abundant myofilament proteins, is the main molecular motor that provides chemical energy for muscle contraction and considered as the marker of myofiber type (Knight & Molloy, 2000). Troponin is a Ca^2+^ binding protein complex (including troponin C, I, and T) and presents in the filament of myofibrils (Marston & Zamora, 2020). Tropomyosin is an α-helix actin-binding protein, which, together with troponin complex, plays an indispensable role in regulating the contraction and filament assembly of both myocardial and skeletal muscle (Marston & Zamora, 2020). In *P. dentex* skeletal muscle, 35 myofibrillar DEGs were found, of which 14 genes were up-regulated in fast-twitch muscle and 21 were up-regulated in slow-twitch muscle (Fig. 8C). The differences in the transcription levels of these genes may lead to differentiation of protein expression as well as physiological properties, and further, induce the skeletal muscle fibers highly specialized.

## Conclusions

In conclusion, we have produced and analyzed in-depth transcriptome data of both fast-twitch muscle and slow-twitch muscle in *P. dentex*, a deep-sea migratory species with potential for artificial breeding. We identified thousands of differentially expressed genes between these two muscle types, which play important roles in the mitochondrion energy metabolism and skeletal muscle structure. Differential expression of key genes involved in lipid and glycogen metabolism pathways confirmed at molecular level that lipid and glycogen are the main energy sources for slow-twitch muscle and fast-twitch muscle, respectively. Numerous key enzymes involved in electron transport and mitochondrial oxidative phosphorylation were significantly upregulated at mRNA expression levels inferred that the slow-twitch muscle had a stronger higher oxidative phosphorylation to ensure more energy supply. Expression signatures of the main skeletal muscle developmental genes indicated more myogenic cells in the differentiating state in fast-twitch muscle. Structural genes may lead to the skeletal muscle fibers specialized were also characterized. The results in this study captured the fundamental physiological and metabolic differences at molecular level between fast-twitch and slow-twitch muscles in *P. dentex*. Furthermore, this work will contribute to the development of deep-sea mariculture objects and the mining of long-distance migratory fish genetic resources.

## Supplemental Information

10.7717/peerj.12720/supp-1 Supplemental Information 1 Statistical of genes in different expression levels.

10.7717/peerj.12720/supp-2 Supplemental Information 2 Expression levels validation by RT-qPCR.

10.7717/peerj.12720/supp-3 Supplemental Information 3 Sequencing and assembly statistics of the genome information of *Pseudocaranx dentex*.

10.7717/peerj.12720/supp-4 Supplemental Information 4 Animal Research: Reporting *I. Vivo* Experiments.

## Additional Information and Declarations

### DNA Deposition

The following information was supplied regarding the deposition of DNA sequences: The raw data of transcriptome sequences are available at NCBI Sequence Read Archive (SRA): SRR14672383, SRR14672384, SRR14672385, SRR14672386, SRR14672387 and SRR14672388. https://dataview.ncbi.nlm.nih.gov/object/PRJNA733284?reviewer=6rd3girmpca3ou03301k1niuat. The accession number for *Pseudocaranx dentex* reference genome is PRJNA731999. The raw sequence data of transcriptome are also available at Figshare with Digital Object Identifier: 10.6084/m9.figshare.15153537, and the link https://figshare.com/s/5e4abfae0eeec2f2e627.

### Data Availability

The following information was supplied regarding data availability: The raw sequence data of transcriptome are available at Figshare: Wang, Huan (2021): The transcriptome sequencing data of *Pseudocaranx dentex* skeletal muslce. figshare. Dataset. https://doi.org/10.6084/m9.figshare.15153537.v1. The raw data for RT-qPCR are available in Table S2.
